# Supplementary material for: Evaluation of Health-Related Quality of Life in Patients with Euthyroid Hashimoto’s Thyroiditis under Long-Term Levothyroxine Therapy: A Prospective Case-Control Study
Source: J Clin Med. 2024 May 24;13(11):3082. doi: 10.3390/jcm13113082 (PMC11173285; doi:10.3390/jcm13113082)
Supplement: Supplementary file 1 [file jcm-13-03082-s001.zip › jcm-2995923-supplementary.pdf]

SF-12 Health Survey

This survey asks for your views about your health. This information will help keep track of how you feel and how well you are able to do your usual activities. **Answer each question by choosing just one answer**. If you are unsure how to answer a question, please give the best answer you can.

1. **In general, would you say your health is**:

☐1 Excellent ☐2 Very good ☐3 Good ☐4 Fair ☐5 Poor

The following questions are about activities you might do during a typical day. Does <u>your health now limit you</u> in these activities? If so, how much?

	**YES, limited a lot**	**YES, limited a little**	**NO, not limited at all**
2. **Moderate activities** such as moving a table, pushing a vacuum cleaner, bowling, or playing golf.	☐1	☐2	☐3
3. Climbing **several** flights of stairs.	☐1	☐2	☐3

During the <u>past 4 weeks</u>, have you had any of the following problems with your work or other regular daily activities <u>as a result of your physical health</u>?

	**YES**	**NO**
4. **Accomplished less** than you would like.	☐1	☐2
5. Were limited in the **kind** of work or other activities.	☐1	☐2

During the <u>past 4 weeks</u>, have you had any of the following problems with your work or other regular daily activities <u>as a result of any emotional problems</u> (such as feeling depressed or anxious)?

	**YES**	**NO**
6. **Accomplished less** than you would like.	☐1	☐2
7. Did work or activities **less carefully than usual**.	☐1	☐2

8. **During the <u>past 4 weeks</u>, how much <u>did pain interfere</u> with your normal work (including work outside the home and housework)?**

☐1 Not at all ☐2 A little bit ☐3 Moderately ☐4 Quite a bit ☐5 Extremely

These questions are about how you have been feeling during the <u>past 4 weeks</u>.
For each question, please give the one answer that comes closest to the way you have been feeling.

How much of the time during the <u>past 4 weeks</u>…

	All of the time	Most of the time	A good bit of the time	Some of the time	A little of the time	None of the time
9. Have you felt calm & peaceful?	☐1	☐2	☐3	☐4	☐5	☐6
10. Did you have a lot of energy?	☐1	☐2	☐3	☐4	☐5	☐6
11. Have you felt down-hearted and blue?	☐1	☐2	☐3	☐4	☐5	☐6

12. **During the <u>past 4 weeks</u>, how much of the time has your <u>physical health or emotional problems</u> interfered with your social activities (like visiting friends, relatives, etc.)?**

☐1 All of the time ☐2 Most of the time ☐3 Some of the time ☐4 A little of the time ☐5 None of the time

Patient name: Date: PCS: MCS:

Visit type (circle one)

Preop 6 week 3 month 6 month 12 month 24 month Other:_________
